# Distributions of four taste and odor compounds in the sediment and overlying water at different ecology environment in Taihu Lake

**DOI:** 10.1038/s41598-018-24564-z

**Published:** 2018-04-18

**Authors:** Heyong Huang, Xiaoguang Xu, Xiansheng Liu, Ruiming Han, Jine Liu, Guoxiang Wang

**Affiliations:** 1 School of Geography Science, Nanjing Normal University, Wenyuan Road 1, Nanjing, 210023 P. R. China; 2 School of Environment, Nanjing Normal University, Wenyuan Road 1, Nanjing, 210023 P. R. China; 3 Analysis and Testing Center, Nanjing Normal University, Wenyuan Road 1, Nanjing, 210023 P. R. China

## Abstract

Organic matter-induced black blooms, such as cyanobacterial and vegetation blooms, are a serious ecosystem disasters that have occurred in Taihu Lake. After large-scale outbreaks of blooms in eutrophic water, a large number of cyanobacterial and vegetation residue accumulate in the coastal areas, and rapidly fermented into odorous compounds. In this study, four taste and odor compounds have been analyzed in sediments and overlying water of different ecology environment in Taihu Lake. High concentrations of DMDS (up to 7165.25 ngg^−1^ dw^−1^), DMTS (up to 50.93 ngg^−1^ dw^−1^), β-cyclocitral (up to 5441.69 ngg^−1^ dw^−1^), β-ionone (up to 1669.37 ngg^−1^ dw^−1^) were detected in sediments. Also, the spatial distributions of DMDS, DMTS, β-cyclocitral and β-ionone in the sediments were investigated. As the depth of sediment increases, nutrients and odorous compounds are greatly reduced. The results showed that during the degradation of cyanobacterial and vegetation residues, DMDS, DMTS, β-cyclocitral, β-ionone and nutrients are gradually released. In addition, when assessing the source of odorous compounds in overlying water, it should also be considered that it may be released from the sediment. This study shows that odorous compounds are ubiquitous in near-shore zones Taihu Lake, and may take potential hazard to aquatic ecosystems.

## Introduction

Massive cyanobacterial and vegetation blooms are a visible ecosystem response to advanced eutrophication^1,2^. However, the decrease in dissolved oxygen (DO) levels in bottom waters that results from the degradation of large amounts of organic matter is regarded as the most serious threat from these blooms^1,3^. Moreover, excessive organic matter in the water column can result in hypoxia, even anoxia, in the water and surface sediments. Hypoxia and anoxia can induce black water bloom disasters in freshwater lakes^4,5^. Because of the degradation of organic matter from cyanobacteria blooms and/or polluted sediments, some of the most important freshwater lakes in China, such as Lake Dianchi, Lake Chaohu, and Lake Taihu, have been suffering from black blooms for many years. These black blooms have drawn the attention of the government and academicians. All the black blooms occurred unpredictably in late spring or early summer, were near the shore, and usually lasted from 24 hr to 2 weeks. Black blooms are identified by the black color of the water and are often accompanied by taste and odor (T&O) compounds.

Therefore, more attentions has been drawn to T&O compounds. In the past years, odorous compounds, such as hydrogen sulfide, methanethiol, 2-methylisoborneol, geosmin and 2-isopropyl-3-methoxypyrazine have been extensively investigated^6,7^. Recently, β-cyclocitral and β-ionone^8,9^, two metabolites of cyanobacteria^10,11^, have been monitored several times in environment^9,12^ and have attracted great attention. Dimethylsulfide (DMS), dimethyldisulfide (DMDS) and dimethyltrisulfide (DMTS), originate from the decomposition of sulfur-containing organic matter are the major components responsible for the strong offensive odor of black bloom^13^. Because these T&O compounds often break out in natural waters simultaneously^7^, investigation of these compounds will provide valuable information to assess their fates in water environment. Until now, there have been numerous research on odorous compounds in drinking water or lakes, reservoirs and rivers^7,14,15^, however, few studies have investigated the distribution of these compounds in sediment and water. Understanding the fates of T&O compounds in different environmental compartments (e.g., sediment, surface water) will be useful to assess the behavior of T&O compounds in aquatic environment.

Lake Taihu, the third largest freshwater lake and important water-source lake in China. Over the past years, algae-induced black blooms have occurred frequently in the west of Lake Taihu and caused serious disasters to ecological environment^16^. Although odor-producing cyanobacteria and odorous compounds have been frequently occurred in surface water of Lake Taihu^17^, odorants in sediment and their potential contribution to the overlying water have not been systematically investigated. We have studied the distributions of two odorous compounds in the overlying water and sediment of the typical sites of Taihu Lake^18^. However, further studies must be extend to more T&O compounds distribution between sediments and overlying water is necessarily. In recently year, continuous ditch digging, reed planting, sludge dredging and so on have increase the environmental complexity of Taihu Lake, which simultaneously influence the production-release-degradation of odorous compounds.

The goal of our research aims to monitor the residues of DMDS, DMTS, β-cyclocitral and β-ionone in the sediment and overlying water off the different environmental condition of Taihu Lake. Specifically, concentration of total nitrogen, total phosphorus, total organic carbon (TOC), nitrate nitrogen, ammonium were investigated. Spatial trends of the physical-chemical parameters were examined to reveal the differences in different zones. The results will be better to understanding the fate, decomposition and release of odorous compounds in the lake aquatic environment.

## Materials and Methods

### Materials

The standard of four odor compounds DMDS, DMTS, β-cyclocitral and β-ionone (100 mg/L, methanol) were purchased from Sigma-Aldrich (Milwaukee, WI, USA), and the standard solutions were diluted with MilliQ-water. Other reagents were of analytical grade and used without further purification.

Solid phase micro-extraction (SPME) coated with stableflex DVB/CAR/PDMS fibers(50/30 μm) was purchased from Sigma-Aldrich (Milwaukee, WI, USA).

### Sample collection

Lake Taihu (30°55′40″–31°32′58″N, 119°52′32″–120°36′10″E), is a typical shallow eutrophic subtropical lake in China. Many serious black bloom incidents have recently occurred in Lake Taihu, especially along the northwestern bay and shorelines.

**Figure 1:**
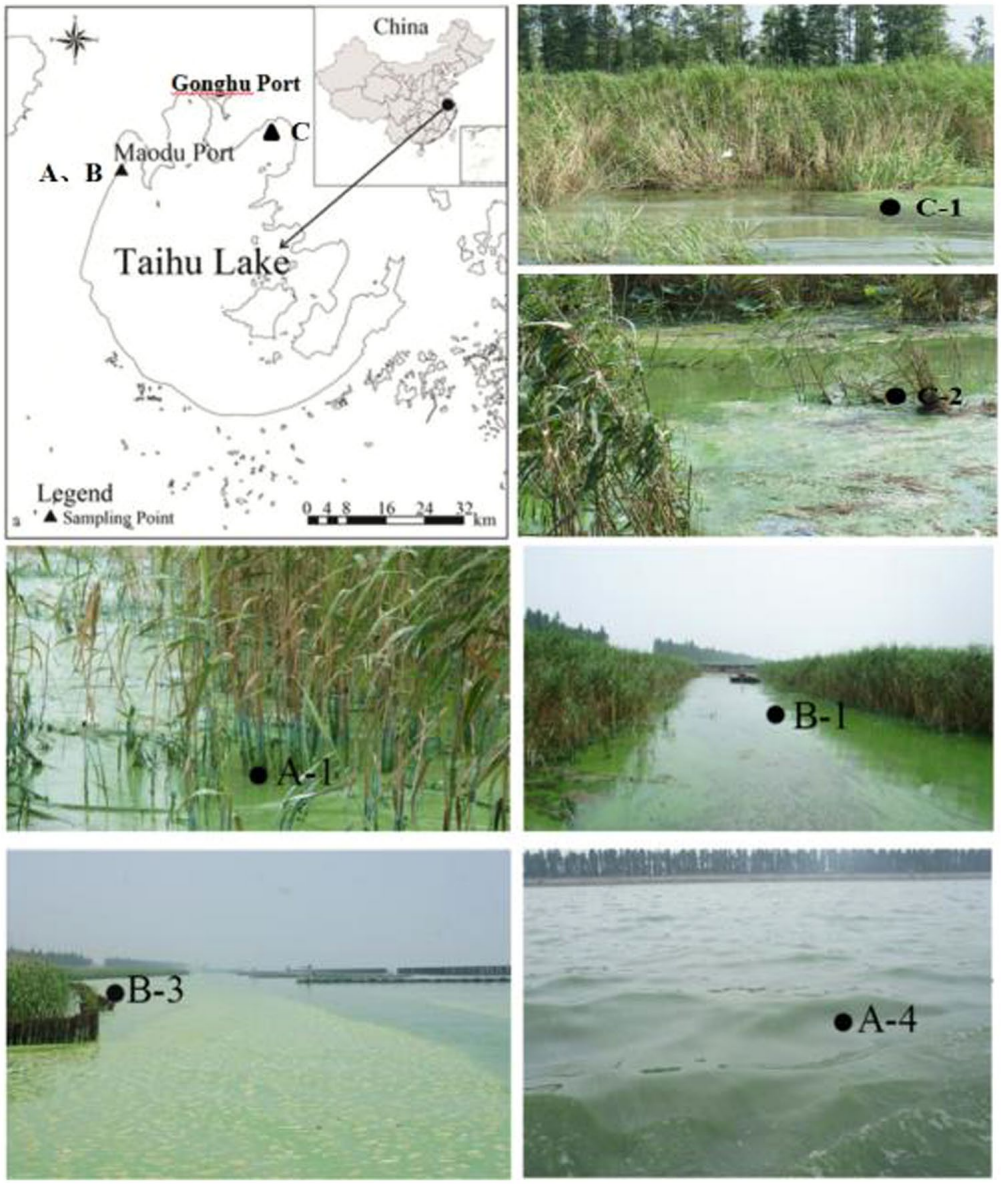
Location of the sampling points in Zhushan Bay and Gonghu Bay, Taihu Lake, China. (Environmental conditions of typical sampling points can be seen in color figures). The figure is created by ArcGIS 10.2 (http://www.esri.com).

Sampling A (A-1 to A-4), B (B-1 to B-4) were collecting in the Zhushan Bay and Sampling C (C-1 to C-4) was collecting in the Gonghu Bay of Taihu Lake, where events of cyanobacterial blooms have occurred frequently in recent years. All sampling points are displayed in Fig. 1, and A-1 to A-4, B-1 to B-4, C-1 to C-4 are from lake shoreline to lake water area. Points A-1 and A-2 are in the natural reed growing area; B-1 and B-2 are located in the man-made reed area while B-3 is close to the reed area, facing the open area; C-1 is near reed area, C-2 is between Potamogeton crispus and reed area, C-3 is near Potamogeton crispus; the other points are all in the open area. The sediment and overlying water samples were collected by gravity columnar dredge. 50 mL of water was quickly separated and stored in amber glass bottles for further analysis. The sediment samples were sliced every 2 cm from top to bottom. Then about 5 g each layers of precipitate was immediately stored into amber glass bottle. Samples prepared for organic carbon and nutrients samples were saved under the dark at −20 °C and 0–4 °C, respectively. Odor compounds analysis were saved in the dark under 4 °C and analyzed within 7 days.

### Analysis of overlying water and sediment samples

Water temperature, dissolved oxygen (DO), pH and oxidation-reduction potential (ORP) were measured *in situ* using a HORIBA water quality monitor (HORIBA, Ltd. Kyoto Japan). Nitrate nitrogen (NO^−^_3_-N) and ammonium nitrogen (NH_4_^+^-N) were determined using an auto-analyzer (Auto-analyzer 3, SEAL, Germany). Total nitrogen (TN) was tested by UV-VIS-NIR spectrometer (Cary5000, Varian, USA)^19^. Total phosphorus (TP) was analyzed using the protocol proposed by Ruban^20^. Samples for total organic carbon (TOC) analyses were was firstly treated with 10% HCl overnight, and then analyzed with a multi N/C analyzer (HT 1300, analytikjena, Germany).

### Determination of four odor compounds

The concentration of DMDS, DMTS, β-cyclocitral and β-ionone were measured by using SPME coupled with GC/MS according to previous studies^15,21,22^.

Extraction was conducted with a DVB/CAR/PDMS (50/30 μm) for 30 min at 65 °C with a stirring speed at 500 r/min. After incubation, the fiber was retracted and injected into the injector of GC-MS (Agilent 7890AGC, 5975MSD, USA) and desorbed in splitless mode for 2 min at 250 °C. The GC column was programmed from 60 °C (constant temperature for 1 min) to 220 °C (10 °C/min, hold constant for 5 min). The EI-MS conditions are as follows: 230 °C for ion-source and 70 eV for ionizing voltage; scan range, m/z 40–350 amu; cycle time, 0.5 s. For the selection of monitoring (SIM) mode, m/z 94 and 79 for DMDS, m/z 126 and 111 for DMTS, m/z 152 and 137 for β-cyclocitral, m/z 177 and 135 for β-ionone were monitored.

### Statistical analysis

All samples were analyzed in duplicate or triplicate. In order to achieve quality control, quality control samples and standard addition samples were also analyzed simultaneously during the analysis process.

The Statistical Package of the Social Science 18.0 (SPSS 18.0) was used for statistical analysis. The one-way analysis of variance (ANOVA) and correlation analysis was carried out using bivariate correlations analysis. The correlation between the nutrients and odorous compounds were developed by linear correlation analysis.

## Results and Discussion

### Analysis of overlying water and sediment samples

The characteristics of the sediments and overlying water can be found in Table 1. Obviously, the sediment collected in the open areas (A-3, A-4, B-3, B-4, C-3 and C-4) was more shallow than in the near-shore zones (A-1, A-2, B-1, B-2, C-1 and C-2), while the depth of water shows the opposite trend. And extremely low pH, DO and ORP can bee found in sites A-1, A-2, B-1, B-2, C-1 and C-2. The lower DO and ORP indicating the strong reducing conditions were formed at water-sediment interface, which might be associated with a large number of reducing bacteria^23^.

**Table 1:** Physical and chemical characters of the overlying water and sediment from the sampling points A, B and C in Taihu Lake.

Point	The Average Thickness of Algae/cm	Temperature/°C	Depth of Water/m	Depth of Sediment/cm	pH Overlying Water	DO/mg/L Overlying Water	ORP/mV Overlying Water
A-1	2	29.1	1.5	18	6.7	1.2	−222.9
A-2	1.5	29	2.3	18	7.4	2.1	−46.9
A-3	0.2	29.4	3.0	28	9.3	14.4	4.8
A-4	0.0	29.9	3.1	4	9.3	12.2	14.5
B-1	2	29.2	1.3	44	6.9	0.5	−214.5
B-2	2	30.3	1.5	38	7.4	4.5	−98.7
B-3	2.5	31.6	2.6	18	9.6	6.1	−64.1
B-4	0.0	31.2	3.1	4	9.0	16.4	8.2
C-1	1.5	29.2	1.0	44	7.26	0.6	96.5
C-2	2	30.4	1.4	23	7.33	2.03	75.3
C-3	0.5	29.6	1.8	15	7.89	6.03	78.0
C-4	0.0	30.4	2.3	12	8.30	7.90	66.2

**Figure 2:**
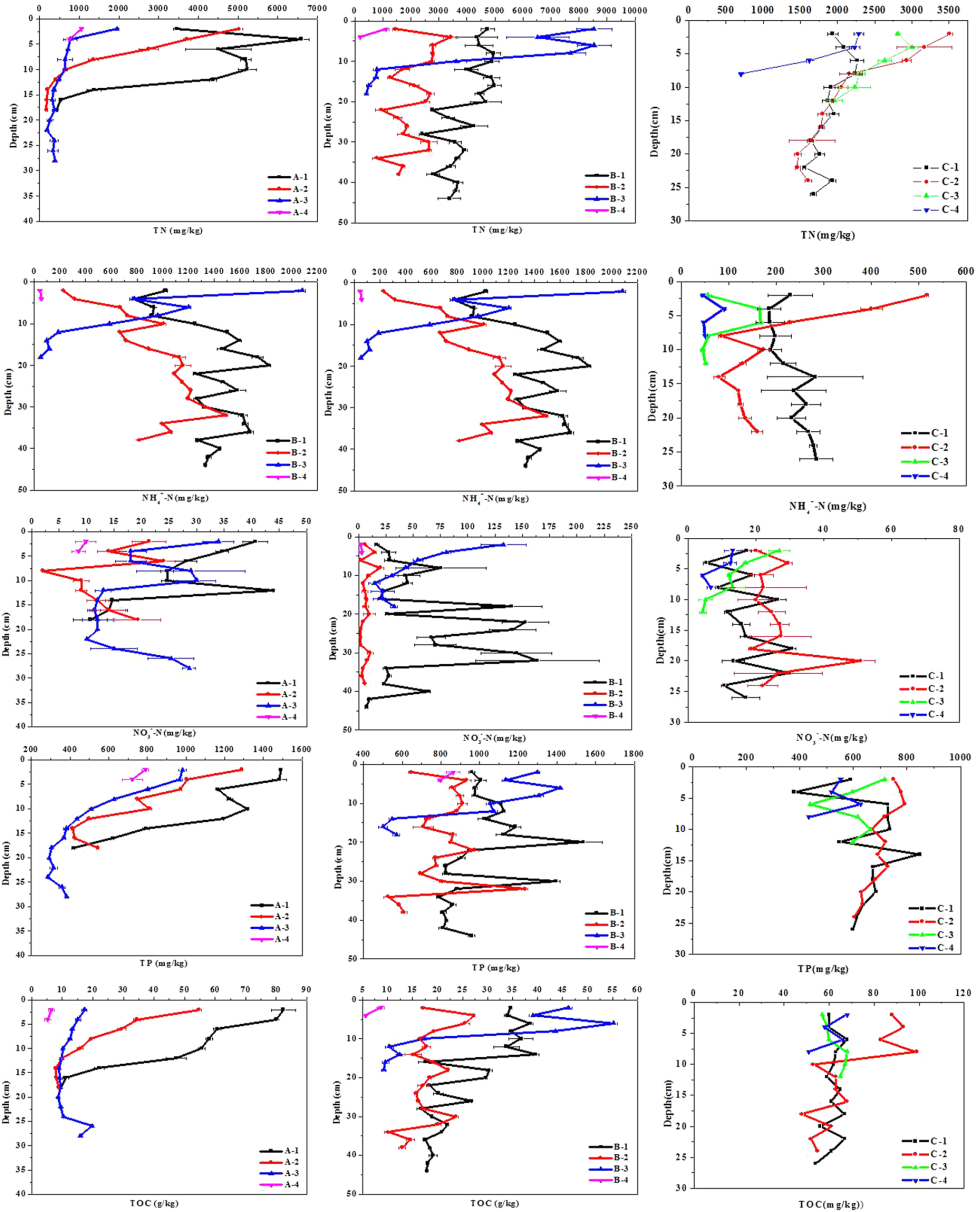
Changing trends of nutrients and TOC in the sediment from the sampling points (**A**,**B** and **C**) in Taihu Lake.

It has been found that excessive organic matter in water may cause hypoxia, even anoxia, in the water and surface sediments. Hypoxia and anoxia can result in black water bloom disasters in freshwater lakes^4,5^. Higher temperature lead to higher microorganism activity, this might consuming more oxygen in summer and further accelerates the mineralization process. The decomposition of algae and vegetation will consume a large amount of DO, which resulted in the release of a huge amount of organic matter and nutrients (Fig. 2). Feng *et al*. have found under the conditions of anoxic or anaerobic, organics can effectively promote the outbreaks of black bloom^23^. They thought that the black bloom may be originate from the co-synergistic metabolisms between organic and micro-organisms in the metabolic sediment.

The concentrations of nutrients and TOC in the sediment can be seen in Fig. 2. In the study areas, the nitrogen concentrations in the sediment range from 204.16 to 8543.68 mg/kg for TN, 1.99–132.87 mg/kg for NO_3_^−^-N and 23.97–2082.71 mg/kg for NH_4_^+^-N, with average values of 2988.91, 24.76 and 420.66 mg/kg, respectively. The phosphorus concentrations varied from 378.91 to 1561.69 mg/kg in the sediment, with an average value of 828.04 mg/kg. And the TOC concentrations varied from 5.15 g/kg to 111. 03 g/kg in the sediment, with a mean value of 50.11 g/kg.

In the vertical distribution, the concentration of nutrients and TOC fluctuated with depth. The values of TN followed by A-1 (4990.91 mg/kg), A-2 (2697.25 mg/kg), A-3 (943.38 mg/kg) and A-4 (908.87 mg/kg), similar phenomenon was found in other nutrients and TOC at section A. However, at section B, the highest concentrations of TN appeared at point B-3, where a large amount of phytoplankto was washed ashore, the same phenomenon was found in other sediment nutrients. However, at section C, the concentrations of TN followed by C-2 (2763.00 mg/kg), C-3 (2595.00 mg/kg), C-1 (2084.15 mg/kg) and C-4 (1709.26 mg/kg), which might contribute to the co-decomposition of submerged plant Potamogeton and cyanobacteria. Overall, in the near-shore zone, the concentrations of nutrients and TOC were higher than those open area zone, the result suggesting that in shallow eutrophic lakes dredging will be an effective method for the reduction of sediment nutrients.

For the spatial distribution, the content of nutrients in point B fluctuate greater than those in point A, C in near-shore area, especially in man-made wetlands. This might be associated with the frequent human activities involving the construction of wetlands. Although with the depth of the sediment increased, the nutrient contents decreased. However, in the ditch, especially at point B-1, the level of nutrient were still high. This might be due to the accumulation of large amount of cyanobacteria in the ditch. Furthermore, nutrient contents in section A are higher than those in section C along the shore, which might be related to the absorption of nutrients by Potamogeton crispus^24^. Finally, the massive accumulation of cyanobacteria and ultimate death resulted in a strong anaerobic reduction environment^12^. In this case, nutrients will be gradually released into the overlying water, resulting in dramatically deterioration of the aquatic environment. Although the nutrients concentrations of section A and B were higher than those in section C, the concentrations of TOC at section C were remarkably the highest in three sections. The co-decomposition of dead Potamogeton plant and cyanobacteria may released more organic matter into the water column and contributed to the higher TOC concentration at section C. Overall, there were significant differences between different ecology environment in their nutrients and TOC contents, such as reed areas, Potamogeton crispus, ditch, and dredged areas.

### Distribution of odorants in overlying water and surface sediment

**Figure 3:**
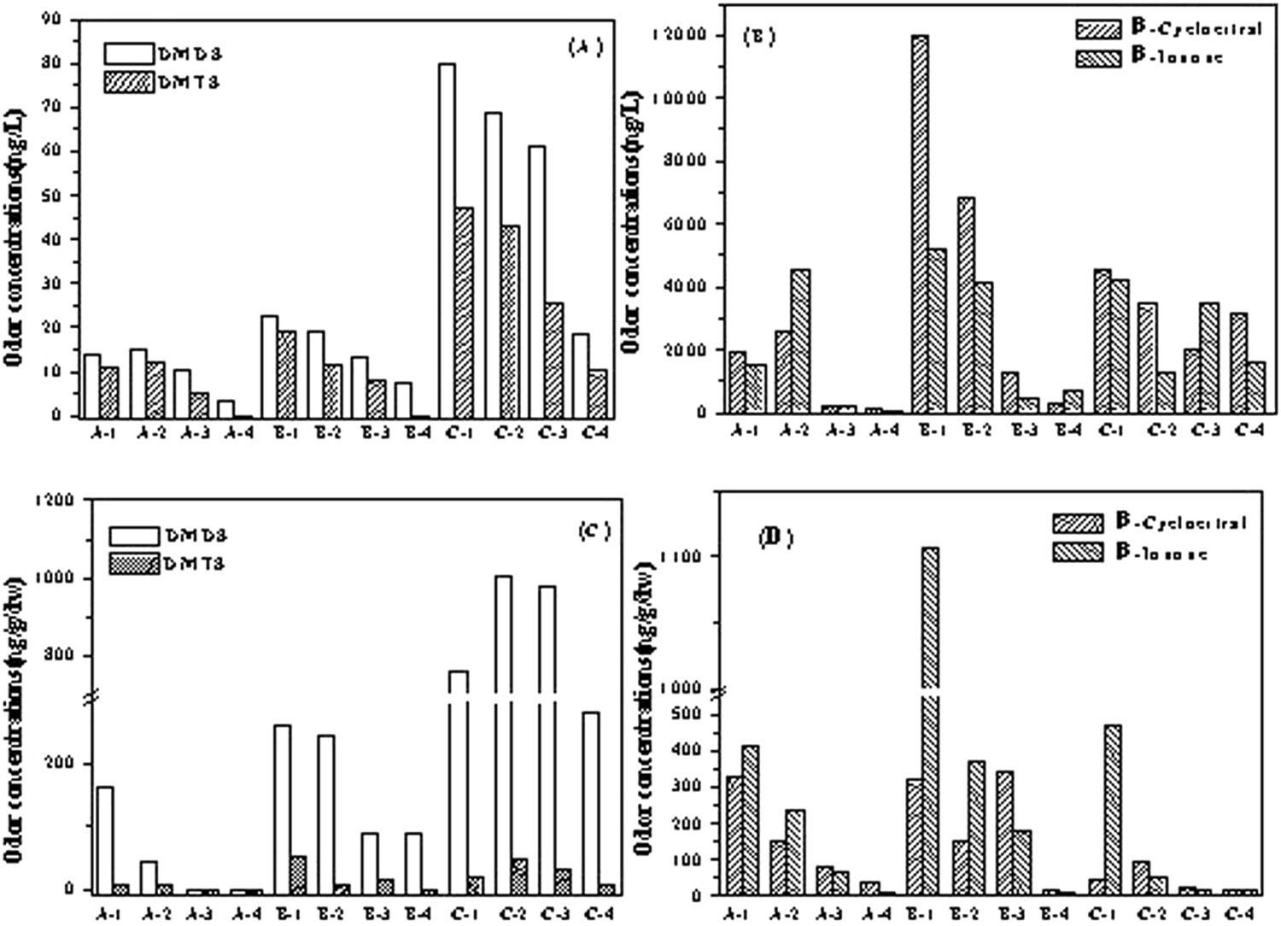
The concentration of four odorants in overlying water (**A**,**B**) and sediments (**C**,**D**) from the sampling points (**A**,**B** and **C**) in TaihuLake.

The spatial distribution of the four odorants in overlying water and sediments in different sampling points can be found in Fig. 3. The high contents of odorants detected in surface sediment probably due to the adsorption of sediments^25^.

The odorants contents in the overlying water varied from 3.29 to 78.83 ng/L for DMDS and nd to 46.88 ng/L for DMTS, with mean values of 28.01 and 16.85 μg/L, respectively. And the contents of odorants in the surface sediment varied from nd to 1003.31 ng/(g•dw) for DMDS and nd to 50.93 ng/(g•dw) for DMTS, with the average value of 336.71 and 15.70 ng/(g•dw), respectively. The variety trends of DMDS and DMTS in the overlying water and surface sediment are similar. The correlation coefficient is 0.897 for DMDS, and the correlation coefficient is 0.849 for DMTS. Based on the Spearman correlation analysis, it was a significant correlation between overlying water and surface sediment at the level of P < 0.05. In this case, odorants present the risk of diffusion from sediment to overlying water^26^. Additional, the odorants detected in the water varied from 150 to 12020 ng/L for β-cyclocitral and 30 to 5160 ng/L for β-ionone, with the average values of 3194.46 and 2083.43 ng/L, respectively. In the surface sediment, the contents varied from 5.40 to 789.92 ng/(g•dw) for β-cyclocitral and 6.97 to 1106.96 ng/(g•dw) for β-ionone, with the average values of 196.91 and 260.03 ng/(g•dw), respectively.

Due to the gradual decomposition of cyanobacteria and vegetation, there were high odorous concentrations in some special area of Taihu Lake. And the distribution features observed in our study resemble the previous studies^27,28^. It was found that the highest DMDS contents appears at the site of C-2 in surface sediment, but not C-1. Also, the highest β-cyclocitral contents was detected in surface sediment at the site of B-3, but not B-1 or B-2. As we know, point B-3 is nearby reed area and faces the open lake area, while site C-2 is between the reed area and Potamogeton crispus. Affected by storms and hot dry weather, large number of fresh cyanobacteria gathered, and then decayed, deposited finally. Eventually, a large amount of organic and odorous matters were gradually released into overlying water and the sediments. Therefore, it might be necessary to prevent the accumulation and decomposition of cyanobacteria for lessen odor issues in Taihu Lake.

### Vertical and horizontal distribution of odorants in the sediments

**Figure 4:**
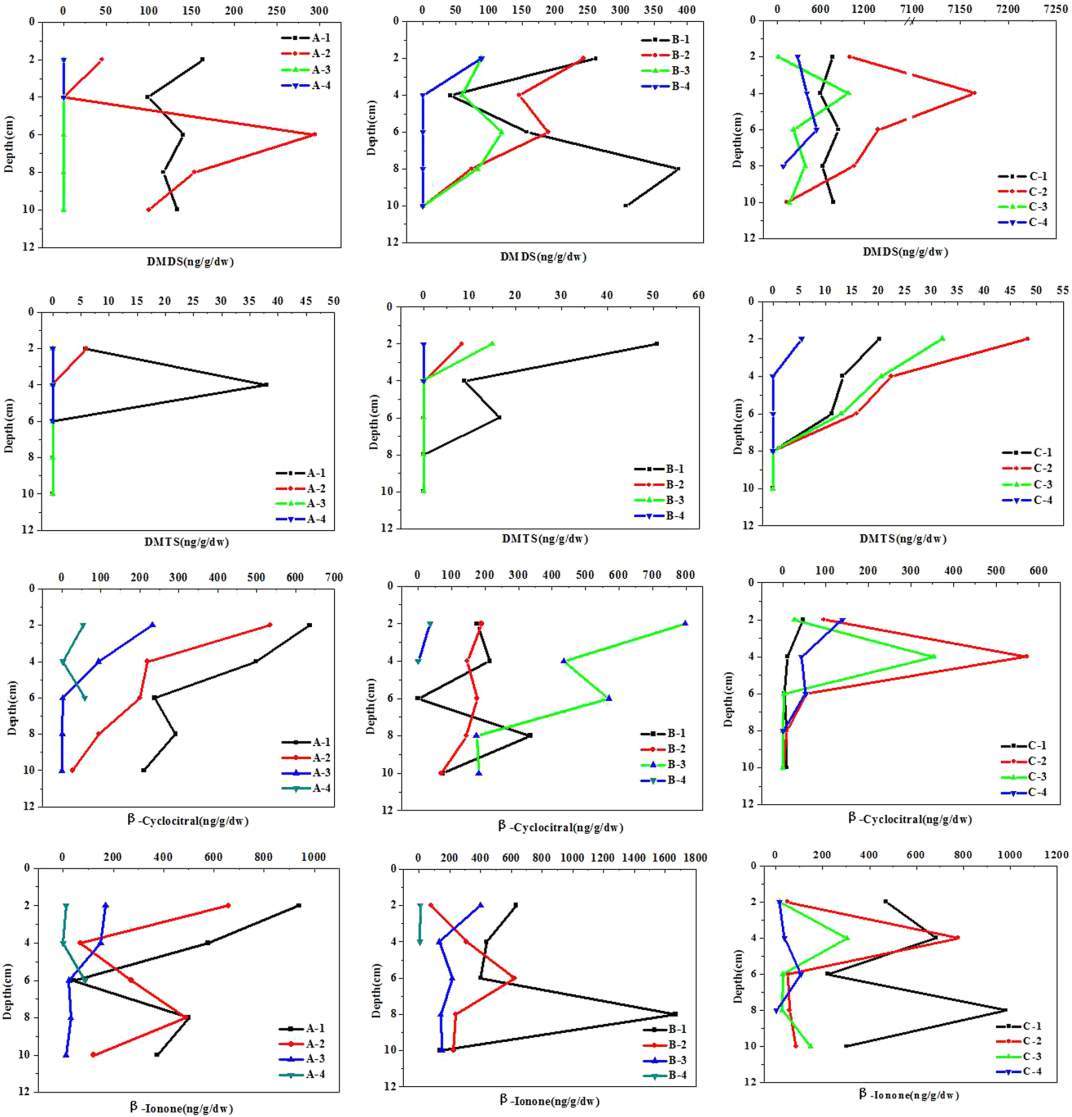
Change trend chart of four odorants in the sediment of points (**A**,**B** and **C**) in TaihuLake.

The vertical and horizontal ditribution of the odorants in the sediments can be found in Fig. 4. The content of odorants shows a large range of fluctuation in sediments, from nd to 7165.25 ng/(g•dw), with the mean value of 388.04 ng/(g•dw) for DMDS, and varied from nd to 50.93 ng/(g•dw) with the mean value of 6.34 ng/(g•dw) for DMTS. The concentrations of DMDS at sites A followed by A-1 (231.28 ng/g), A-2 (130.63 ng/g), A-3 (69.94 ng/g) and A-4 (29.54 ng/g), and similar distribution characteristics can be found at site B. The highest concentration of DMDS was found at site C-2 (2150.97 ng/g). The concentrations of DMTS at sites A followed by A-1 (8.76 ng/g), A-2 (1.19 ng/g), A-3 (nd) and A-4 (nd). The highest content of DMTS was also detected at site C-2 (17.34 ng/g). It was reasonable to assume that the high-density cyanobacteria accumulated in water then gradually deposited on surface sediment. As we know, the decomposition of the vegetation and cyanobacteria will consume large amount of DO, eventually leading to the realse of a large amount of organic matters. Volatile organic sulfur compounds in freshwater lakes are produced primarily by phytoplankton and alga Metabolism or microbial degradation of organic matter^29,30^. The anaerobic decomposition of cyanobacteria and vegetation can promote DMDS and DMTS production in point A and B. The highest production of DMDS and DMTS at section C may resulted from the decomposition of sulfur-containing organic compounds in dead Potamogeton and cyanobacteria, as this process generates a variety of methylated sulfides^6,30–32^.

Also, the concentration of β-cyclocitral in sediments fluctuated from 0.11 to 5441.69 ng/(g•dw), with the mean value of 365.06 ng/(g•dw), and from 0.11 to 5441.69 ng/(g•dw) with the mean value of 259.08 ng/(g•dw) for β-ionone. The content of β-cyclocitral at sites A followed by A-1 (375.17 ng/g), A-2 (215.36 ng/g), A-3 (65.79 ng/g) and A-4 (38.68 ng/g). And at site C followed by C-2 (146.39 ng/g), C-3 (76.52 ng/g), C-4 (59.07 ng/g) and C-1 (15.55 ng/g). The highest content of β-cyclocitral was detected in point B-3. The concentrations of β-ionone at sites A followed by A-1 (486.76 ng/g), A-2 (322.15 ng/g), A-3 (76.52 ng/g) and A-4 (32.22 ng/g), and similar distribution characteristics can be found at site B and C. The highest content of β-ionone can be seen at site B-1 (655.45 ng/g).

The odorants content showed clearly downward trend in the sediment (<4 cm) along open area (A-3, A-4, B-4 and C-4), and the concentration are all far below that in the coastal zone. No doubtly, this difference can be owing to the different environment conditions and to the accumulation of cyanobacteria. A one-way ANOVA was applied to analysis the differences among odorant compounds. We thought that high content of odorants existed in the sediments may originated from the decomposition of cyanobacteria owing to the climate and topography. As reported, influenced by prevailing winds, cyanobacteria can easily gather in the bay and western area of the Taihu Lake, especially on the shores of the Zhushan Bay, Meiliang Bay and Gonghu Bay^33^. Previous studies have shown that the type of enon-wetland-dyke or intermittent-wetland-dyk wetland area will influence the drifting and aggregation of algal^34^. Of course, the shoreline and ditch embayments will also promote the accumulation of algae.

Compared with the previous research (shown in Table 2), in water, the mean value of β-cyclocitral was about 6 to 28 times than the concentration in the other regions. And the content of β-ionone have the similar phenomenon, the mean value of β-ionone in water of our research was 10 to 41 times than that other regions. Simultaneously, the mean value of β-cyclocitral in the sediment of Zhushan Bay was about 7 to 182 times than other regions. And β-ionone concentration was more than 9 to 259 times that in the other regions. Surprisingly, the mean value of DMTS were lower than that in Poyanghu Lake^21^.

**Table 2:** Taste and odor compounds in other areas.

Odorants	Mean Concentrations in water	Mean Concentrations in sediment	Area	Source
DMDS	1100 ng/L	—	Gonghu Bay in Taihu Lake	Shen *et al*.^41^
	**27.79 ng/L**	**388.04 ng/g**	Taihu Lake	This study
DMTS	69.55 ng/L	—	Gonghu Bay in Taihu Lake	Chen *et al*.^33^
	—	4 ng/g	Chaohu Lake	Deng *et al*.^21^
	—	51 ng/g	Poyanghu Lake	Deng *et al*.^21^
	—	3 ng/g	Taihu Lake	Deng *et al*.^21^
	**16.06 ng/L**	**6.34 ng/g**	Taihu Lake	This study
β-Cyclocitral	537.61 ng/L	—	Gonghu Bay in Taihu Lake	Chen *et al*.^33^
	115 ng/L	—	Dianchi Lake	Li *et al*.^27^
	—	2 ng/g	Chaohu Lake	Deng *et al*.^21^
	—	49 ng/g	Poyanghu Lake	Deng *et al*.^19^
	—	18 ng/g	Taihu Lake	Deng *et al*.^21^
	**3209 ng/L**	**365 ng/g**	Taihu Lake	This study
β-Ionone	50.44 ng/L	—	Gonghu Bay in Taihu Lake	Chen *et al*.^33^
	200 ng/L	—	Dianchi Lake	Li *et al*.^27^
	—	1 ng/g	Chaohu Lake	Deng *et al*.,^21^
	—	259 ng/g	Poyanghu Lake	Deng *et al*.^21^
	—	29 ng/g	Taihu Lake	Deng *et al*.^21^
	**2282** **ng/L**	**259 ng/g**	Taihu Lake	This study

### Correlation between C/N ratios, nutrients, TOC and odorants

Carbon to nitrogen ratios (C/N) have been widely applied to recognize the sources of organic matter, as we know, the C/N ratios of higher plant-derived organic matter (C/N > 20) are much higher than the corresponding ratio of plankton and bacteria (6–7 and 4–5, respectively)^35,36^. In the near-shore area, the C/N ratios of A-1 and A-2 were 34.63 and 39.86, followed by site C-1 (31.05), C-2 (25.08), C-3 (20.29) and B-1 (7.64), B-2 (8.01), B-3 (5.77), respectively. The values of C/N in site A,C were higher than those of site B, indicating that a large amount of bacteria and plankton gathered at point B.

The correlation between the odorous compounds and the C/N ratios in sediment are displayed in Table 3. Zuo *et al*. have revealed that in Xionghe Reservoir, geosmin was positively correlated with TN, TOC and chlorophyll-a in the sediment^15^. As we know, nutrients are favor cyanobacteria blooms, and the growth of cyanobacteria was limited by nitrogen and phosphorus during the growing season^2^. Therefor, the nutrients can regulate the production of odor compounds directly or indirectly^37,38^. Figures 2 and 4 and Table 3 revealed that there were similar varying trends of odorants and nutrients (TN, NH_4_^+^-N, TP and TOC) in sediment. Yang *et al*. considered that the black water “agglomerate” in Taihu Lake was related to the high NH_4_^+^-N levels^5^. In our study, both β-cyclocitral and β-ionone showed good correlations with NH_4_^+^-N. Meanwhile, DMDS and DMTS were more closely related to TOC in the sediment, while β-cyclocitral and β-ionone were more closely related to nitrogen in the sediment.

**Table 3:** Correlation between the available nutrients and the taste and odor compounds.

Index	TN	NH_4_^+^-N	NO^−^_3_^–^N	TP	TOC	C/N	DMDS	DMTS	β-cyclocitral	β-ionone
TN	1									
NH_4_^+^-N	0.734**	1								
NO^−^_3_^–^N	0.569**	0.373**	1							
TP	0.605**	0.615**	0.349**	1						
TOC	0.320**	−0.029	0.225	−0.218	1					
C/N	−0.450**	−0.537**	−0.247*	−0.643**	0.632**	1				
DMDS	0.063	−0.098	−0.062	−0.309*	0.722**	0.612**	1			
DMTS	0.348**	0.136	0.116	0.002	0.485**	0.139	0.333**	1		
β-cyclocitral	0.671**	0.502**	0.432**	0.441**	0.261*	−0.352**	0.109	0.323**	1	
β-ionone	0.492**	0.640**	0.237	0.290*	0.180	−0.177	0.266*	0.253*	0.493**	1

## Conclusions

This research shows the occurrence of four odorous compounds DMDS, DMTS, β-cyclocitral and β-ionone off the bay of Taihu Lake. The results shows that odorous compounds were ubiquitous in overlying water and sediment.

The vertical distribution of nutrient, organic matters and odorous compounds in sediment reveal that there were significant differences among the different ecology environment types. Reed roots not only absorb nutrients but also absorb odorous compounds, this indicating that planting reeds probably a viable method to reduce the production of odorous compounds. However, the massive degradation of dead Potamogeton crispus may increase the concentration of nutrients and odorous compounds, this indicate that the planting of submerged plants should be under suitable density.

In addition, the strong decomposition of cyanobacterial residues in typical topography under suitable climate, can release a large amount of odorous compounds and cause the malodor associated with black blooms. As the high odorous compounds detected in sediment, sediment dredging is a feasible method for the reduction of nutrients and odorous compounds originate from the sediment to the overlying water. Therefore, combination sediment dredging with preventing cyanobacteria from aggregating may be the most viable strategy to suppress odor ecological problem in eutrophic shallow lakes^39,40^.
